# Healthcare professionals’ experiences of de-labelling erroneous penicillin allergy records in general practice: a qualitative study

**DOI:** 10.3399/BJGPO.2024.0119

**Published:** 2025-07-16

**Authors:** Caity Roleston, Marta Santillo, Kelsey F Armitage, Catherine E Porter, Shadia Ahmed, Joanne Fielding, Marta Wanat, Christopher C Butler, Sue Pavitt, Jonathan AT Sandoe, Sarah Tonkin-Crine

**Affiliations:** 1 Nuffield Department of Primary Care Health Sciences, University of Oxford, Oxford, UK; 2 Leeds Institute of Medical Research, School of Medicine, University of Leeds, Leeds, UK; 3 Leeds Teaching Hospitals NHS Trust, Leeds, UK; 4 Dental Translational and Clinical Research Unit, School of Dentistry, University of Leeds, Leeds, UK; 5 NIHR Health Protection Research Unit in Healthcare Associated Infections and Antimicrobial Resistance, University of Oxford, Oxford, UK

**Keywords:** qualitative research, allergies, hypersensitivity, primary health care

## Abstract

**Background:**

Penicillin allergy (PenA) prevalence is approximately 6%, but fewer than 10% of these people are expected to be truly allergic. Consequently, a significant proportion of the population are prescribed alternative antibiotics with potential increased risk of acquiring multi-drug resistant bacteria and worse health outcomes. The ALlergy AntiBiotics And Microbial resistAnce (ALABAMA) trial aimed to determine if a penicillin allergy assessment pathway (PAAP) initiated in primary care, is effective in de-labelling erroneous records, improving antibiotic prescribing and patient outcomes.

**Aim:**

To investigate healthcare professionals’ (HCPs’) experiences of the ALABAMA trial.

**Design & setting:**

Qualitative study using semi-structured interviews in general practice in England.

**Method:**

Semi-structured interviews were conducted with HCPs (including GPs, research nurses, pharmacists) who delivered the trial. Interviews explored their views about de-labelling incorrect PenA records, their role(s) in the trial, and, where relevant, their experience of prescribing following de-labelling.

**Results:**

HCPs (*n* = 18) believed many patients were incorrectly labelled PenA, and were aware of the individual and public health risks this posed. However, GPs explained labels were rarely challenged in general practice because the perceived risks to patients and their professionalism were too great. The PAAP intervention, alongside the ‘protocolisation’ within the ALABAMA trial, was successful at mitigating these risks. Consequently, the trial was well-accepted and commended by HCPs.

**Conclusion:**

GPs welcomed and accepted the PAAP as a means of correcting erroneous PenA records. There is great potential for PAAP to be supported in primary care if testing becomes more accessible.

## How this fits in

There is currently little evidence on healthcare professionals’ (HCPs’) views and experiences of supporting a penicillin allergy assessment pathway (PAAP) to de-label erroneous penicillin allergy (PenA) labels. This article adds to the knowledge base. It found GPs were aware of the risks associated with incorrect PenA labels but rarely challenged them owing to perceived risk to patients and their professionalism. A PAAP intervention, alongside the ‘protocolisation’ within the ALABAMA trial, was successful at mitigating these risks. The implications for clinicians are that there is great potential for PAAP to be supported in primary care if testing is accessible and if integrated care boards commission GPs to refer eligible patients.

## Introduction

Approximately, 6% of the UK population have a record of penicillin allergy (PenA),^1^ but fewer than 1 in 10 people who think they are allergic have a true allergy.^2–4^ Incorrect PenA labels have important implications for both individual and public health. Patients labelled as penicillin allergic usually do not receive optimal standard-of-care antibiotic treatment or prophylaxis. Patients with PenA experience longer hospital stays, increased admission to intensive care units, and increased mortality when compared with people without a PenA record.^1,3,5^ Additionally, they are at higher risk of re-prescription of a new antibiotic class within 28 days,^1^ receiving a second-choice antibiotic,^6^ and dental implant failure.^7^ These patients are prescribed broad spectrum antibiotics more often, putting them at increased risk of acquiring multi-drug resistant bacteria, such as methicillin-resistant *Staphylococcus aureus* (MRSA), and *Clostridioides difficile,* ^5,8^ compared with people without an allergy record.

Primary care is responsible for 80% of antibiotic prescribing in the UK^9^ and between 2021 and 2022, penicillin prescribing in primary care increased by 23%, owing in part to increased group A streptococcal infection as COVID-19 social distancing guidelines were relaxed.^9^ If we are to meet the demands of the UK’s national plan to optimise the use of antimicrobials,^10^ it is necessary to spotlight antimicrobial resistance and re-prioritise stewardship efforts across the healthcare system, but in particular within primary care. Given the consequences of incorrect PenA labelling, it is incumbent on healthcare providers to improve the pathway(s) that enable patients with PenA identified as low risk of allergy to be screened and tested, with a view to de-label those without a true allergy.^11^

A recent qualitative study in the UK demonstrated that while GPs often doubted the veracity of their patients’ allergy status, few challenged the label for fear of ’making a mistake*‘* that caused patient harm or prompted legal action against them.^12,13^ Additionally, there was a lack of consensus over what symptoms (outside of anaphylaxis) GPs considered to be indicative of an allergic reaction, which made diagnosing PenA, and differentiating true allergy from drug side effects, challenging.^12^ Furthermore, a rapid literature review examining patient and prescriber views of penicillin allergy testing (PAT) and subsequent antibiotic use^14^ found patient referral for formal allergy testing by healthcare professionals (HCPs) happened infrequently across countries (which included Canada, France, the UK, and the US). The most frequently encountered barriers among GPs were, anticipated resistance from patients, not having time within the consultation to discuss testing, and a lack of availability of allergy testing services.

There is a paucity of evidence concerning implementation and evaluation of PenA de-labelling interventions in primary care. A primary care pathway study, where GPs identified patients with PenA label and referred them to a secondary care testing service, found patients felt the process was useful.^15^ While the sample size was small (*n* = 37), none experienced a reaction during drug provocation testing, and most (90%) indicated they would take penicillin in the future after testing negative. A GP survey within the programme indicated the greatest challenges to recruitment were patients not wanting to be tested and time constraints. While there is an emerging interest in primary care-focused PenA de-labelling interventions,^15–17^ the experiences and views of GPs participating in such interventions are not well understood as studies rarely capitalise on qualitative methodologies.

**Figure 1.**
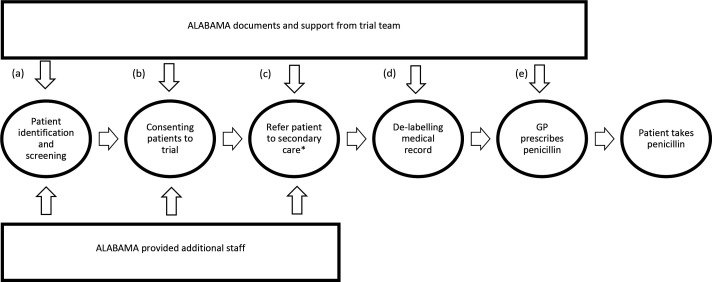
ALABAMA Penicillin Allergy Assessment Pathway (PAAP). (a) Site training and working instructions; ’Penicillin Allergy Testing: information for general practice’ leaflet; (b) working instructions; (c) working instructions; (d) allergy result letter; ‘task’ sent to GP in SystmOne electronic health record (EHR) system; (e) electronic health record pop-up^22^. *Those randomised to intervention arm. Note: Healthcare professionals were purposively recruited to ensure we spoke to people with experiences across each stage in the trial, that is, from (a) to (e).

The ALlergy AntiBiotics And Microbial resistAnce (ALABAMA) trial aimed to determine if an intervention package, centred around a penicillin allergy assessment pathway (PAAP) (see Figure 1) initiated in primary care, was effective in de-labelling erroneous records, and improving antibiotic prescribing and patient outcomes.^18^ General practice patient records were reviewed to identify adults with a PenA label but considered ‘low risk’ (no history of anaphylaxis or other severe allergic reaction) (see also, Table 1). Patients who met these eligibility criteria were invited to participate in the trial. Consenting participants were randomised to receive either usual clinical care or were referred for penicillin allergy assessment. Based on history and a risk stratification process, participants received either a skin test followed by an oral challenge test or a direct oral challenge test. All participants who did not react to the first doses, were also directed to continue the oral challenge test for a further 3 days at home. Both patients and their general practice were informed of the test result by letter, the results were also sent electronically via electronic health records. In cases where the result was negative, general practices were instructed on how to remove the PenA label from their patient’s record.

**Table 1.** ALABAMA guidance for completing patient eligibility form

ALABAMA guidance for completing patient eligibility form
a) If the patient answers ’Don’t know, can’t remember, or not sure’ to any of the questions in section 3 of the eligibility form, please tick the ’NO’ box. Please confirm the absence of an allergy history consistent with anaphylaxis to penicillin; for example, patient reports anaphylaxis or symptoms compatible with anaphylaxis such as collapse, difficulty breathing, loss of consciousness, or swelling of the face or tongue or lips (not including transient tingling sensation in lips or tongue). Patients who report isolated facial swelling without systemic symptoms may still be eligible and should be discussed with the trial team. Patients with brittle asthma or severe asthma (see [trial resource pack] for definitions) are not eligible, patients without brittle or severe asthma who have had recent steroids (in the past 3 months) can still be consented once steroid free for 3 months if their asthma is well controlled. Patients are ineligible if they are unable to omit beta-blocker medication on the day of penicillin allergy testing. Patients are ineligible if they are currently taking antihistamines or medication with antihistamine properties* and are unable to stop these 3 days pre-testing.** *Please refer to [trial resource pack] for list of drugs with antihistamine properties. **Some patients who are taking medication with antihistamine properties, for example, amitriptyline, might still be eligible and should therefore be discussed with the ALABAMA team before consent (see contact details below).
b) Once you are 100% sure that the patient is eligible (all the answers to the inclusion criteria on the form must be **Yes** and all the answers to the exclusion criteria must be **No** ), click submit if you are using Sentry or sign the eligibility form if you are using the paper copy and you can then start the informed consent process (consent process and systems instructions provided in document).
c) If any of the answers to the exclusion criteria are **Yes**, do not move on to the informed consent process. If you are unsure if the patient is eligible or you have any queries when carrying out the eligibility check, you can contact the ALABAMA Research Fellow (contact details provided).

There is limited qualitative research examining the experiences of HCPs participating in primary care-led PenA de-labelling interventions. This study sought to respond to this gap by exploring HCPs’ views and experiences of the ALABAMA trial (which included PAAP, trial procedures, and implications of de-labelling on subsequent antibiotic prescribing and penicillin use). We aimed to leverage these unique insights to inform future research and practice in the field.

## Method

### Design

Qualitative study using semi-structured interviews in general practice and secondary care in England.

### Recruitment

HCPs were purposively recruited to ensure we captured data relevant to each stage in the trial; from patient identification, screening and consent, to secondary care testing, de-labelling, and antibiotic prescribing (where relevant). To achieve this, email invitations were sent to all GPs participating in the ALABAMA trial as well as to HCPs who consented patients to the trial and those who performed the drug provocation test in secondary care sites. Additionally, GPs who had prescribed antibiotics to ALABAMA patients who had tested negative were identified and invited to interview. All prospective participants were emailed a copy of the participant information leaflet and a consent form and asked to contact the first author if they were interested in participating.

### Interviews

A topic guide was developed based on the primary research questions and informed by the ALABAMA feasibility study.^19^ The topic guide centred around the views of participants on referring patients for testing, testing processes, de-labelling, and prescribing penicillin (to patients who had tested negative), and the intervention materials provided. The guide remained flexible, which permitted us to follow the participant’s agenda (Supplementary file).

Verbal consent was obtained from participants before interviews were conducted. Interviews were conducted online by an experienced qualitative researcher (PhD qualified with substantial previous experience conducting qualitative health research), were audio-recorded and transcribed verbatim by an independent transcription company. To preserve anonymity, participants are referred to using participant number and job role (for example, P1, GP).

### Analysis

Data collection and analysis were performed concurrently. Data from all interviews were analysed. Transcripts were read and reread by CR both during and after data collection. Data were analysed using inductive thematic analysis.^20^ To enhance the rigour of our analysis, initial transcripts (*n* = 4) were read and analysed by the wider team (CR, MS, ST-C) to ensure data were accurately represented and to agree on preliminary codes. Based on this discussion, CR developed a coding framework, which was applied to the rest of the dataset with changes made if needed. This preliminary coding framework elicited ‘themes’ that more accurately represented ‘domain summaries’, that is, they largely mirrored the topic guide questions and the chronological processes within the trial.^20^ After further discussion as a group (CR, MS, ST-C), we identified the related concepts of ‘risk’ and ‘risk mitigation’ to be an overarching theme that helped us understand participants’ experiences on a deeper level. CR then returned to the data using the lens of ‘risk’ to develop the themes presented here. Together, the three themes present HCPs’ journeys through the ALABAMA trial. First, by concentrating on their pre-trial experiences of suspected erroneous PenA labels and the challenges they face managing them without access to timely allergy testing services. Followed by attending to the ways in which the ALABAMA intervention and trial responded to, and ameliorated, these challenges.

## Results

### Participants

Of the 54 HCPs invited to participate in this study, 18 (33%) completed an interview. Of these, 13 were GPs, two were research nurses who consented patients to the trial, and three (2 = pharmacist, 1 = research nurse) performed drug provocation testing. The sample consisted of HCPs from general practices in Cornwall and across Yorkshire (reflecting the regions participating in the trial). Interviews were conducted between August 2023 and February 2024; this was towards the end of the trial when no more patients were being recruited. Interviews lasted 22–49 minutes (mean: 36 minutes).

First, it is noted, HCPs were encouraged to reflect on their experiences of each aspect of the trial (for example, identification of patient records, screening and eligibility, consent and randomisation, allergy testing, de-labelling, prescribing), including any challenges or areas for improvement. Participants were unanimous, and emphatic, in their praise for the trial, *‘I think this is probably the best study we’ve ever been involved with’* (P2, GP), and areas for improvement hinged on individual preferences (for example, video instructions for de-labelling in addition to written instructions). While we agree that *‘you don’t often hear the positives, do you; you just hear the negatives, so it is important to hear that, too’* (P1, GP), we elected to present findings that speak not only to participants’ views on the various trial processes, but also deepens our understanding of how the trial elicited behaviour change.

We present three themes that explore HCPs’ views on the reasons that led to the high prevalence of erroneous PenA labels, the patient and professional risks inherent in challenging labels without allergy testing, and finally, the ways in which ALABAMA mitigated these risks.

### GPs’ beliefs about why patients have been incorrectly labelled: ’You just can’t make heads nor tails of it’

GPs reflected on the high prevalence of erroneous PenA labels and the ways in which these historical clinical decisions instil their current patient prescribing decisions with risk.

GPs believed they had *‘a fairly significant number of patients’* (P1, GP) who were incorrectly coded with PenA, often because side effects from penicillin-based medication or symptoms from their illness had been conflated with allergy, typically during childhood. However, as the primary event occurred years, often decades ago, the patient’s medical file did not provide their current GP with any context or rationale for the clinical judgement behind the PenA label:

*‘But that’s* [PenA label] *based on a clinical decision and you have no idea about the clinician’s decision-making process, who made that decision, when it was made, maybe fifteen, twenty years ago.’* (P4, GP)

GPs explained this uncertainty was amplified among patients whose PenA label predated the digitisation of medical files as there was a paucity of detail in the paper records, or else they were illegible, *‘the paper records you just can’t read and there’s no detail (…) you just can’t make heads nor tails of it’* (P2, GP). This paucity of information made it challenging for GPs to assess whether the patient had a true PenA.

A smaller number of GPs also reflected on the ways in which GP understanding about penicillin had changed over time and that GPs themselves had conflated medication side effects with allergy in the past. One GP noted that their predecessors could not, or did not, anticipate the risks associated with such a liberal approach to PenA labelling based on scant clinical information, *‘documentation of penicillin allergy became an incredibly important thing and no one considered this could possibly have a downside to it’* (P11, GP). They went on to argue that all GPs were therefore *‘complicit’* in the preponderance of incorrect PenA labels in general practice.

Ultimately, GPs felt that there was no clear guidance on how to challenge a patient record with a historically ambiguous PenA label, or those presenting with non-anaphylactic reactions to a medication, *‘*[if] *they have anaphylaxis then yes, we will refer to the allergy clinic, but apart from that there’s no clear guidance to these allergy reactions’* (P8, GP). Consequently, GPs rarely, if ever, challenged patients’ PenA label and continued to avoid penicillin-based medications in favour of convenient second-line alternatives, *‘if there’s any doubt, we would just go with the alternative’* (P2, GP).

### Penicillin allergy de-labelling without testing: ’If we’re feeling very, very brave, we might do a trial’

Indeed, most GPs reported being *‘very cautious’* about *‘trialling’* penicillin among those labelled as allergic. Some explained they *‘occasionally, if we’re feeling very, very brave, we might do a trial and see and get them to let us know if they were okay with it’* with the caveat that *‘that’s probably quite rare’* (P2, GP). However, others were much more reticent, *‘so most clinicians will be reluctant to prescribe against a coded allergy, if that makes sense. I probably don’t even question it very much’* (P4, GP).

The risk to patient safety when conducting *‘trials’* was the primary concern for GPs as they were *‘in the community with no backup’* (P14, GP) and feared the patient may experience (fatal) anaphylaxis. This was particularly poignant in one general practice that had a history of patient fatality attributable to PenA:

*‘A long time ago we had a patient who suffered an allergic reaction to a penicillin and died because of that and that shadow is hanging over us, it’s more than 20 years since this incident (…)* [but] *we’re very risk averse.’* (P16, pharmacist)

One GP also emphasised the personal risk required to initiate such trials:

*‘You know, we live in an environment where we have a vengeful regulator (…) no-one’s got any confidence in the GMC* [General Medical Council] *at all, that they’re not just gonna* [sic] *smash you. And so, everyone sort of feels in fear of their professional registration.’* (P11, GP)

GPs also felt patient beliefs about their medical history, particularly if they had been labelled as penicillin allergic for many years, would lead to reticence about trying penicillin or being de-labelled without undergoing allergy testing:

*‘A lot of patients that think they’re allergic, that have been told for forty years that they’re allergic, are very resistant when you then say to them, "actually, if it was just that you had a tummy upset, that isn’t really an allergy"’.* (P1, GP)

These risks notwithstanding, some GPs went on to discuss the conditions required to remove PenA labels without drug provocation testing. First, GPs reported that if the patient’s medical history revealed they had previously been prescribed and tolerated a penicillin, this would be sufficient for them to remove the label, *‘I mean sometimes you do have patients who say "well, I had a different penicillin a year ago and I didn’t have any reactions" and if I can see that then I will take allergies off, I will remove it’* (P7, GP). In such cases GPs interpreted this historical tolerance of penicillin as an indicator the patient was not truly allergic and they were therefore confident that a dangerous reaction was unlikely.

Second, in order to open up the idea of trialling, GPs emphasised the importance of *‘drilling down into the* [patient’s] *record’* to better understand the context of the primary event that gave rise to the label, and any subsequent events, *‘you say "well what actually happened?" and "oh when I was a child my mum said I just got sick with this one" and then you think, well, actually that’s probably not an allergy’* (P9, GP). GPs explained that this then opened up a *‘discussion with the patient’* (P3, GP) about their treatment options, which necessitated getting *‘them* [the patient] *to help assume some of the risk as well, like get them agreed towards a trial of that particular antibiotic if it was only a very mild allergy’* (P3, GP). But again, GPs emphasised theirs was an inherently cautious profession, and in practice, it was rare that ambiguous PenA labels were challenged.

### Ways ALABAMA mitigated risk: ’Being [in a] protocolised and a protected supported bubble; makes you much safer to do it’

This theme examines the ways in which risk was mitigated through participation in the ALABAMA trial. Central to this theme was a guidance document, developed by the trial team, which enabled HCPs to check patient eligibility, obtain informed consent, and complete electronic referral to the trial where participants would then be randomised. HCPs were also reassured that each patient would be assessed at multiple timepoints within the trial (that is, eligibility screening, referral to PAAP) using these robust tools. It was through this, and other supporting guidance documents, that clinicians felt confident to identify low-risk patients, recommend allergy testing and refer them to the trial, and later, de-label patients who had tested negative.

As an exemplar, the guidance document provided to HCPs to aid patient eligibility screening has been reproduced in Table 1, but note, this is but one section of a larger instructional document.

Across all participants a central strength of the trial was that it was *‘totally protocolised (…) so once you’ve got the allergy history, you’re not left working out is that low risk? or high risk? (…) there’s a flow chart that leads you to the right answer’* (P17, pharmacist). Embedded within this ’algorithm*‘* was a referral pathway to more senior clinicians for ambiguous or contradictory cases, *‘some were not straightforward so I had to speak to the clinician to find out if they were potentially eligible’* (P19, research nurse). While this could be frustrating as it delayed patient recruitment, particularly in sites where patients could not recall their ‘allergic reaction’ or reported vague symptoms such a rash, most were grateful for this added rigour in the consent procedure.

Additionally, the procedures for identifying and assessing patient risk were repeated at key points throughout the trial, including the identification of patients from primary care records, consenting patients, and at the test clinic. Again, this provided HCPs with assurance that only patients identified as *‘low risk’* would be tested, *‘patients have been stratified by the time they get to us, the GP’s gone through their record and so we’re only getting the low-risk patients anyway’* (P16, pharmacist, test clinic). HCPs felt confident that patients were being *‘properly tested in a safe environment’* (P2, GP) and emphasised that *‘as medics, we work in an environment where we test all the time, and if somebody devises a test to check for penicillin allergy and it comes back negative, you’ve got to trust that, haven’t you?’* (P6, GP).

Clinicians at test centres reported patients had been *‘well prepared during the early parts of the study’* (P16, pharmacist) but nevertheless *‘we probably went through in quite detail about what the testing would involve*’, particularly as it related to whether patients would go straight to oral challenge test or have skin testing first. Consequently, few patients registered any questions or concerns about the test procedures or their personal risk being tested, and most accepted whichever testing pathway they were offered. Among the very small number of patients who HCPs reported had anxiety about being tested, most were reassured by clinicians’ two-pronged approach to first explain only those who are *‘very low risk get involved in the trial’* and second, *‘you are coming to a clinical environment which is very controlled’*, and well equipped to manage emergency situations should they arise (P18, research nurse).

Four of the GPs in the study had consulted with patients who had been de-labelled as part of the trial. They characterised prescribing penicillin-based medications as unproblematic, *‘I have prescribed antibiotics which were penicillin based (…) and they* [the patient] *were pretty happy with it. They didn’t question me about it. They took it*’ (P14, GP). Those without direct experience hypothesised they *‘wouldn’t hesitate to decide that’s the best antibiotic for them’* (P6, GP). Some GPs said they would spend additional time with patients to ensure they understood the change in their allergy status, were well informed about the potential side effects of the medication, and that they *‘were happy’* (P6, GP).

## Discussion

### Summary

This study explored HCPs’ views of erroneous PenA labels and their experiences delivering a PAAP intervention. Discussions with HCPs about the provenance of incorrect PenA labels indicated that GPs regularly see patients with PenA labels, which they believe to be incorrect, but incomplete medical histories and poor patient recall introduce doubt. This uncertainty made it difficult to determine whether a patient was truly allergic and most reported they did not routinely challenge PenA labels. Some GPs reported challenging patients’ PenA status when they were feeling ’*very brave*’ but most felt it was ‘*too risky*’ — both for the patient and themselves — to conduct drug provocation testing with patients in primary care without appropriate support and guidance.

In practice, the PAAP intervention and ALABAMA trial functioned to mitigate risk for GPs and other allied HCPs. Central to this was the ’protocolisation’ of the trial that guided HCPs through the processes and procedures at every step. HCPs were reassured that decisions to identify and refer low-risk patients to PAAP were not theirs alone to make and any potential errors would be identified by colleagues downstream. Lastly, HCPs felt confident in the safety of testing procedures and believed in the accuracy of drug provocation testing. Consequently, GPs were comfortable de-labelling patients who had tested negative, and among the minority who had met with an ALABAMA patient post-test, all described prescribing penicillin as unproblematic.

### Strengths and limitations

To our knowledge, this is the first PenA de-labelling intervention in primary care that has utilised in-depth qualitative interviews to better understand HCPs’ views of the intervention and delivery of a PAAP within a trial. This study builds on our pilot work^19^ to respond to this gap in the literature.

However, the trial was limited in how many general practices enlisted (*n* = 51) and therefore the denominator for our sampling. Additionally, although we purposively identified GPs who had prescribed antibiotics to ALABAMA patients who had tested negative, this strategy did not lead to successful recruitment. Consequently, only one-third of the HCPs invited to participate took part in this study, of which only four GPs had seen a patient who had been de-labelled. While HCPs were recruited from general practices in Cornwall and across Yorkshire (reflecting the regions participating in the trial), by the nature of convenience sampling, only those with the time and inclination participated in the study. The results should therefore be extrapolated with caution.

### Comparison with existing literature

These findings concur with our previous assertions^19^ that GPs have limited experience referring patients to formal allergy assessment but demonstrate a willingness to do so if the service were available and eligibility criteria clear. During the feasibility trial^19^ GPs indicated their confidence reassuring and referring patients for assessment hinged on their beliefs regarding the safety of testing within a hospital environment. The findings presented here go further, such that the ’protocolisation’ of the ALABAMA trial leveraged HCP confidence through processes of shared decision making and responsibility importantly supported by co-created patient literature on PAT and meaning of PAAP test results. This mirrors findings from a UK hospital-based study, which found that protocols and policies made clinicians feel supported and protected to deliver PenA de-labelling.^21^ In short, the (perceived) risks inherent for GPs considering challenging PenA labels — both for patients and themselves — were mitigated by the trial ’protocolisation’.

### Implications for research and practice

Our study has identified important clinical implications. First, GPs believed that increased awareness regarding the prevalence of true penicillin allergy, improved skill in differentiating between side effects and allergy reactions, and the digitisation of medical records would reduce the prevalence of erroneous allergy records over time. However, GPs felt there is currently no clear guidance on how to challenge a patient record with a historically ambiguous PenA label or those presenting with non-anaphylactic reactions to a medication. So how to treat these patients outside of the ALABAMA trial remained a grey area.

Further delineation is also warranted here. GPs spoke extensively about the role suboptimal patient records played in the prevalence of erroneous PenA labels. Specifically, they argued the paucity of information regarding the ‘primary reaction’ complicated their clinical decision making regarding the veracity of the patient’s allergy status and typically resulted in accepting the PenA label without challenge. However, there is a distinction to be made between patients for whom there is good information about their primary event but a lack of evidence they experienced a genuine allergic reaction, in which case GPs should challenge the label, and patients for whom there is little or no evidence regarding their primary event, which would warrant assessment. GPs are not making these distinctions, which suggests that clearer guidance and reassurance is required to identify and differentiate between patient risk profiles and initiate appropriate care pathways (that is, direct de-labelling, referral for allergy assessment).

HCPs reported very little pushback from patients at any stage of the PAAP intervention. They explained they were confident in communicating PAAP procedures and patient risks and benefits. Additionally, they reported patients were motivated to know whether they had a genuine PenA, understood and accepted the PAAP procedures and de-labelling, and among GPs who had seen a PAAP patient who had tested negative, all reported patient(s) had accepted penicillin prescription. Indicative that within this context, the PAAP intervention was accepted by patients.

Finally, the ALABAMA trial provided GPs with a lot of support (for example, step-by-step guidance for how to identify low-risk patients to refer to trial, in-depth instruction on de-labelling, and near same-time email support for troubleshooting problems), which reduced the perception of risk they felt during usual practice. Consequently, identifying low-risk patients and referring them for allergy assessment became acceptable behaviours. The allergy testing accounts for much of this risk and this is (arguably) replicable in clinical practice. But the trial itself provided extra reassurance (trial funded and run by credible organisations and GPs instructed what to do step-by-step), which may need to be supported instead by integrated care boards (ICBs).

GPs recognise erroneous PenA records as a problem and are supportive to act within their abilities to correct these. They require step-by-step guidance to identify low-risk patients and want a safe, expert service to which to refer patients. GPs are happy to prescribe penicillin once patients have been de-labelled. There is great potential for a de-labelling pathway to be supported in primary care if testing can be accessible to patients and if ICBs can support GPs to refer eligible patients.

## Data

It is not possible to share the data set publicly.
